# Napsin-A Expression, a Reliable Immunohistochemical Marker for Diagnosis of Ovarian and Endometrial Clear Cell Carcinomas 

**DOI:** 10.30699/ijp.2020.106598.2222

**Published:** 2020-02-28

**Authors:** Fatemeh Nili, Mansoureh Tavakoli, Narges Izadi Mood, Hana Saffar, Soheila Sarmadi

**Affiliations:** 1 *Department of Pathology, Imam Khomeini Hospital Complex, Tehran University of Medical Sciences, Tehran, Iran*; 2 *Department of Pathology, Mohebb-e-Yas Women Hospital, Tehran University of Medical Sciences, Tehran, Iran*

**Keywords:** Clear cell carcinoma, Diagnosis, Endometrium, Ovary, Napsin-A

## Abstract

**Background & Objective:**

Clear cell carcinomas (CCC) differ from other types of ovarian and endometrial carcinomas in biology, behavior and response to chemotherapy. Histopathologic diagnosis may be challenging in some situations which necessitates immunohistochemistary (IHC) assessment. In this study we investigated the diagnostic utility of Napsin-A in diagnosis of ovarian and endometrial CCCs.

**Methods:**

Ovarian and endometrial CCC samples from 2013 to 2018 in 3 general and women’s hospital in Tehran were re-evaluated by 2 expert pathologists. Forty-two samples were included as case and 42 non-clear cell carcinomas (Non-CCC) of ovary and endometrium were selected as control group. Based on IHC study tumors with sum intensity and percentage score ≥2 (at least 1+ staining in more than 1% of tumor cells) were considered positive.

**Results:**

The prevalence of endometrial and ovarian CCC in the case group were 15 and 27 respectively. The tumors in the control group included 22 cases of endometrioid, 2 high grade papillary serous carcinoma (HGSC) of endometrium, 6 endometrioid and 12 HGSC of ovary. Napsin-A positivity was observed in 35 (83%) of CCCs while 7 (17%) samples including 3 out of 15 endometrial and 4 out of 27 ovarian CCCs were Napsin-A negative. No positive reaction was seen in control group. The overall accuracy, specifity and sensitivity of Napsin-A for diagnosis of ovarian and endometrial CCCs were 83%, 100% and 83%, respectively. Sensitivity for ovarian and endometrial CCCs were 85% and 80%, orderly.

**Conclusion:**

Napsin-A is an accurate and reliable marker for distinction of CCCs from non-CCCs in ovary and endometrium. A panel of antibodies may yield the highest diagnostic accuracy.

## Introduction

Clear cell carcinoma (CCC) accounts for approximately 10% of ovarian and less than 5% of endometrial epithelial carcinomas (1,2). In ovary, the biology and clinical behavior of ovarian clear cell carcinoma (OCCC) is different from other epithelial tumors. Unlike high grade papillary serous carcinomas (HGSC), OCCC affects younger women in association with endometriosis and is frequently diagnosed in early stages (3). When adjusted for stage, OCCC has the worst prognosis in comparison with other epithelial carcinomas (4,5). Efficacy of platinum-based chemotherapy regimens is between 20 and 50% for OCCC compared with 60 to 80% for HGSC (3). In endometrium, clear cell carcinomas involve older patients and are diagnosed in higher stage of disease with worse prognosis compared with endometrioid or type I carcinomas (2,6). Diagnostic histopathological findings are similar in ovary and endometrial clear cell carcinomas (ECCC). In most of the tumors, a mixture of solid, papillary and tubulo-cystic architectural patterns are seen. The lining epithelial cells are hobnail and/or cuboidal with clear and/or eosinophilic cytoplasm. Mitotic activity is variable but is usually low. High nuclear grade is infrequent (7). Although in most of the cases, histopathological findings are distinctive and characteristic, other types of endometrial and ovarian carcinomas can harbor clear cell changes and make diagnostic confusion (8,9). In a study by Han *et al.*, moderate agreement was observed in classification of the endometrial carcinomas with clear cell changes between the reviewers, where only 46% of original ECCCs were accurately diagnosed (10). 

In this study we evaluated the diagnostic utility of Napsin-A by immunohistochemistry (IHC) for ovarian and endometrial clear cell carcinomas.

## Materials and Methods

**Fig. 1:**
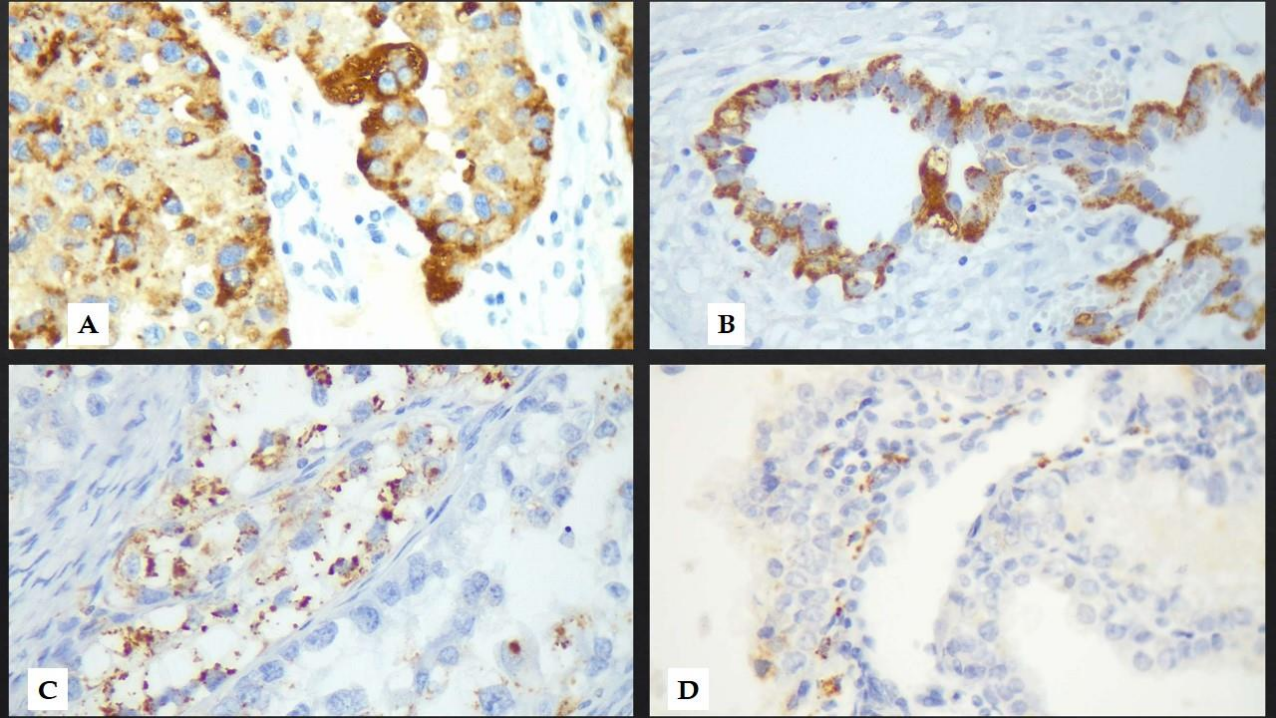
(A and B) IHC stained slides (400x) showing strongly positive coarse (1+) cytoplasmic granules (3+), (C) moderate coarse and fine cytoplasmic granules (2+) and (D) fine cytoplasmic granules

This study was conducted in Imam Khomeini Hospital Complex, Tehran, Iran. Due to scarcity of clear cell carcinoma cases, we designed a case-control study. The study was approved by the Ethics committee of Tehran University of medical Sciences (IR.TUMS.IKHC.REC.1397.304). After reviewing the pathology reports from 2013 to 2018 in 3 general and women’s hospitals in Tehran, ovarian and endometrial clear cell carcinomas were re-evaluated by 2 expert pathologists. Forty-two CCC samples were included as case and 42 non-clear cell carcinomas (non-CCC) in ovary or endometrium were selected as control group. A 4μm thick section from relevant paraffin blocks were taken on charged slides and dried 40 minutes at 60ºC. After deparaffinization and rehydration, heat induced epitope retrieval was performed using Master Diagnóstica EDTA buffer (pH: 6) for 40 minutes at 37ºC. After that, the slides were rinsed with 3-5 changes of distilled water followed by cooling at room temperature for 20 min. Peroxidase solution was used, 10 minutes at room temperature for blocking endogenous peroxidase. After incubation for 60 minutes with primary antibodies for Napsin-A (Master Diagnóstica; Spain), Master Polymer Plus Detection System (HRP) was used for 20 minutes. Finally, the slides were counterstained with haematoxylin and mounted. The IHC scoring was performed based on the intensity of staining (0: negative, 1+: faint cytoplasmic granular, 2+: moderate granular, 3+: coarse cytoplasmic granular positivity) and percentage of positive tumor cells (0: no tumor cell, 1+: 1-25%, 2+: 25-50%, 3+: ≥50%). Tumors with sum score ≥2 were considered positive (Figure 1).

Data analysis was performed using SPSS 22.0 (IBM Inc., Chicago, Illinois, USA). Independent-Sample t-test and Chi-square were used for comparison of continuous variables and nominal and categorical variables respectively. The P-values less than 0.05 were considered statistically significant. Sensitivity, specifity and overall accuracy of Napsin-A were calculated using 2×2 tables.

## Results

Of the CCC samples, 15 were ECCC and 27 samples were OCCC. Of the 42 non-CCC, 22 were endometrioid, 2 were papillary serous carcinoma of endometrium, 6 were endometrioid and 12 were HGSC of ovary. Mean age of the patients in CCC and non-CCC groups were 52.1 years and 57.3 years respectively (*P*=0.08). Other clinicopathologic features including pathologic stage, myometrial invasion, ovarian surface involvement, omental involvement, lymph node metastasis and the difference between CCC and non-CCC cases were summarized in Table 1.

On IHC study, 35 (83%) of CCCs showed positive reaction for Napsin-A. Seven samples (17%) including 3 out of 15 ECCC and 4 out of27 OCCC were negative for Napsin-A. None of the non-CCC samples showed positive reaction for Napsin-A. The overall accuracy, specifity and sensitivity of Napsin-A for diagnosis of ovarian and endometrial CCCs were 83%, 100% and 83%, respectively. Sensitivity for OCCC and ECCCs were 85% and 80%, orderly.

In most of the cases with positive reaction, moderate to severe intensity of staining in more than 25% of the tumor cells were identified. 

No significant statistical difference was identified in ovarian surface involvement, pathologic stage, omental involvement and lymph node metastasis in CCCs with and without immunostaining for Napsin-A (*P*>0.05).

**Table 1:** Clinicopathological features of different ovarian and endometrial carcinoma subtypes in this study

Clinicopathologic features	Clear cell carcinoma	Endometrioid carcinoma	Papillary serous carcinoma	P-value
Histologic type				
Pathologic stage				
pT1 pT2 pT3	22 12 8	22 4 2	3 4 7	0.59
Ovarian surface				
Involved Ruptured Free	16 8 3	1 0 5	11 0 1	0.017
Myometrial invasion				
Less than 50% More than 50%	7 8	13 9	0 2	0.45
Omental involvement				
Involved Free	5 25	3 16	10 4	0.02
Lymph node metastasis				
Present Absent	8 18	0 20	2 8	0.02

**Table 2:** Review of previous studies evaluating diagnostic accuracy of Napsin-A in ovarian and endometrial clear cell carcinoma

Specificity	Sensitivity	Site	Sample size	Year	Author
98%	88%	endometrium	54(CCC), 49(EC), 17(PSC)	2014	Fadare (19)
92.3% ovary 95.5% endometrium	95.5% ovary 66.7% endometrium	Ovary and endometrium	22(CCC ovary), 15(CCC endometrium), 74( Non-CC)	2015	Iwamoto (20)
100%	83%	ovary	86(CCC), 13( clear cell adenofibroma), 101 (Non-CC)	2015	Yamashita (21)
100%	81.3%	ovary	16(CCC), 20(Non-CC)	2016	Sayar (14)
84.2%	66.7%	endometrium	6(CCC) 70(Non-CC)	2017	Al-Maghrabi (22)
89.7%	100%	ovary	25(CCC), 75( Non-CCC)	2018	Hanan (23)
90.9%	100%	0vary	58(CCC), 90 (Non-CCC)	2018	Rekhi (24)
100%	85% ovary 80% endometrium	Ovary and endometrium	42(CCC[ 27 ovary, 15 endometrium]) 42(Non-CCC)	2019	Our study

## Discussion

Endometrial carcinoma is the most common gynecologic cancer in developed countries (11) and ovarian cancer is the fifth frequent tumor in women (12). Clear cell carcinoma is an uncommon subtype of malignant epithelial tumors in ovary and endometrium. Its biology, molecular features, clinical behavior and response to chemotherapy is different from other kinds of carcinomas, which necessitates its accurate diagnosis. Despite histopathological findings, the diagnosis may be challenging and IHC study is crucial. Similar to serous and endometrioid carcinomas, CCCs express CK7, B72.3 and BerEP4 while CA125 and vimentin are positive in approximately 50% of the cases. ER, PR and WT1 are typically negative in CCCS but expression of p53 and p16, which are intermediate between serous and endometrioid carcinomas, is variable (13). Hepatocyte Nuclear Factor-β (HNF-β) is positive in 90 to 100% of CCCs but its specifity is only 54%. Alpha-methylacyl-Coa Racemase (AMACR) is a specific (specifity 99%) but less sensitive (sensitivity 63%) marker for CCC (14). 

Napsin-A is a cytoplasmic aspartic protease, which is predominantly expressed in lung and kidney. In the lung, Napsin-A is expressed in type II alveolar pneumocytes and is involved in the synthesis of surfactant protein. In the kidney, Napsin-A is expressed in proximal tubules and is involved in lysosomal protein catabolism (15). Napsin-A is a well-established new marker for pulmonary adenocarcinoma and is considered to be superior to TTF-1 (16). Its expression in gynecologic CCCs and renal neoplasms has been the subject of some recent studies (14,15,17-24). 

In the present study we found 100% specifity and 83% sensitivity for Napsin-A by IHC, for distinction of OCCC and ECCC from non-CCCs. Our results are in concordance with previous studies, Table 2. Since CCCs are uncommon, we conducted a case-control study to participate a higher number of CCC cases. Inability to determine prevalence, positive predictive and negative predictive values is the limitation of our study. But as we know, case-control studies are superior to cross-sectional studies when the prevalence of the target disease is low and the diagnostic test is costly to perform (25). The results of our study were similar to the findings of the study performed by Yamashita *et al.*, which included the largest sample of OCCCs (86 samples) and non-CCC (101 samples) compared with other studies (21). 

The role of Napsin-A in OCCC and ECCC is not clear. In lung and kidney, expression of Napsin-A is reduced in high grade tumors with advanced clinical stages, therefore a tumor suppressor role has been proposed for this protein (23). Along with some other previous studies, we could not find a significant statistical difference between pathologic stage, omental involvement and lymph node metastasis in negative and positive samples. Hence larger scale studies are needed for this subject.

Fadare *et al.* recommended a panel of immuno-histochemical markers including Napsin-A, HNF-1 βand AMACR to obtain the highest sensitivity and specifity when CCC is a diagnostic consideration (26). 

## Conclusion

Napsin-A is a sensitive and highly specific marker for ovarian and endometrial clear cell carcinoma.
